# Enhanced laboratory surveillance study of Campylobacter species in England

**DOI:** 10.1099/jmm.0.002017

**Published:** 2025-06-12

**Authors:** Craig Swift, Adam Crewdson, Yung-Wai Chan, Anais Painset, Amy Douglas, Suzanne Gokool, Claire Jenkins, Gauri Godbole

**Affiliations:** 1 UK-Health Security Agency, London, England, UK

**Keywords:** *Campylobacter*, enhanced, surveillance, whole-genome sequencing

## Abstract

**Introduction.** Campylobacteriosis is the leading cause of gastroenteritis worldwide, and *Campylobacter* species are the most frequently reported zoonotic, bacterial foodborne pathogens in England.

**Gap statement.** Currently, less than 2.0% of *Campylobacter* isolates in England undergo strain identification and typing, resulting in limited insight into their molecular epidemiology.

**Aim.** To assess the feasibility of using high-throughput whole-genome sequencing (WGS) to generate data for microbiological and epidemiological analysis by the implementation of a 3-month enhanced laboratory surveillance for *Campylobacter* spp. in England, and to make recommendations for improving the current *Campylobacter* surveillance strategies.

**Methodology.** All diagnostic laboratories in England were encouraged to refer isolates of *Campylobacter* spp. for WGS over a 3-month period (7 June–31 August 2021).

**Results.** Over 6,000 *Campylobacter* species isolates were characterized, of which 87.5% were successfully identified as *Campylobacter jejuni* and 8.1% as *Campylobacter coli*. Just over half of the isolates were referred from patients who were male (53%), and *C. coli* isolates tended to be from older patients than *C. jejuni*, with median ages of 55 and 44 years, respectively. The most common multi-locus sequencing type clonal complex identified was ST-21, and within this, the sequencing type ST6175 was the most frequently identified, of which 96.8% were predicted to carry antimicrobial resistance determinants, inferring reduced susceptibility to both ciprofloxacin and tetracycline. The four largest *C. jejuni* 5-single nucleotide polymorphism (SNP) clusters, associated with the larger clonal complexes and sequence type groups (ST6175, ST48, ST6175 and ST5136), accounted for 23.8% (*n*=1,150/4,838) of SNP typable isolates. Conversely, 28.4% and 39.5% of isolates *C. jejuni* and *C. coli,* respectively, appeared to be sporadic, with each isolate assigned a unique SNP address at the 5-SNP level.

**Conclusion.** WGS enabled identification of genetically related clusters of *Campylobacter* isolates in almost real time and shows potential for monitoring of inferred antimicrobial resistance. However, unlocking its full potential requires referral of sufficient and representative isolates for sequencing with parallel epidemiological data collection.

## Introduction

Campylobacteriosis typically presents as self-limiting gastroenteritis characterized by diarrhoea or dysentery, fever and abdominal pain, lasting for 2–10 days. Antibiotic treatment is reserved for more severe cases or when the patient may be at risk of complications, such as the young, the elderly or the immunocompromised [1]. Complications in vulnerable groups of patients include bacteraemia, sepsis and long-term post-infectious autoimmune conditions, such as arthritis, irritable bowel syndrome and Guillain–Barré syndrome (GBS) [2]. There are more than 30 species belonging to the *Campylobacter* genus, although human cases of *Campylobacter* infection are almost exclusively caused by *Campylobacter jejuni *and * Campylobacter coli *[3].

*Campylobacter* species colonize a wide range of wild and domesticated host species, which are capable of surviving within the environment and throughout the entirety of the food chain. They can be transmitted to humans via direct contact with animals or their environment or via the consumption of contaminated food. *C. jejuni* and *C. coli* are frequently isolated from poultry and are considered commensal bacteria in chicken [4]. A more recent, long-running study of retail poultry in the UK carried out by the Food Standards Agency (FSA) showed that *Campylobacter* was detected in 56.0% of whole fresh chickens and that high levels (>1,000 c.f.u. per gram chicken neck skin) were found in 7.0% of those tested (data for 2017 [5]). Alongside their prevalence in chicken meat, *C. jejuni* and *C. coli* are commonly isolated from other retail meats such as beef, pork and lamb [6]. Additionally, infections repeatedly arise via unpasteurized milk [7,9], and untreated water tainted by wild bird or animal faeces also presents a considerable source of campylobacteriosis and has resulted in many large outbreaks all over the world [10,12].

*Campylobacter* is the leading cause of human foodborne bacterial gastroenteritis worldwide. It is the most frequently reported foodborne illness in the European Union (EU), with over 246,000 human cases annually [13,14]. However, the actual number of cases is believed to be closer to nine million each year. In addition to the complications and post-infectious sequelae associated with campylobacteriosis, the sheer prevalence of the disease in developed countries means that the human and economic costs are sizeable. The cost of campylobacteriosis to public health systems and to lost productivity in the EU is estimated by the European Food Safety Authority (EFSA) to be around €2.4 billion a year. In the UK, the costs to patients and the health service alone have been estimated to be £50 million, with an additional £1.26 million caused by subsequent GBS hospitalization, excluding long-term disability costs [15].

Diagnostic laboratories in England have a statutory duty to notify the UK Health Security Agency (UKHSA, formerly Public Health England, PHE) of all detections of *Campylobacter* spp., with over 50,000 reports of *Campylobacter* spp. made to the UKHSA national laboratory surveillance system (Second Generation Surveillance System, SGSS) each year [16]. Although *Campylobacter* is the most common bacterial cause of gastrointestinal infection in the UK, less than 2.0% of isolates detected within England are referred to the Gastrointestinal Bacteria Reference Unit (GBRU) for strain identification and typing. There is, therefore, limited high-quality data on strain characterization and understanding of the molecular epidemiology of *Campylobacter* spp. causing disease in England.

Monitoring of the notification trends for each GI pathogen across the coronavirus disease 2019 (COVID-19) pandemic period highlighted a dramatic increase in diagnoses of campylobacteriosis in the spring of 2021 [17]. To investigate this, UKHSA conducted a 3-month study of enhanced laboratory surveillance for *Campylobacter* spp. in England, using whole-genome sequencing (WGS) to microbiologically characterize isolates recovered from symptomatic patients and referred to the national reference laboratory (GBRU). This study aimed to assess the feasibility of high-throughput sequencing, describe and analyse the molecular typing data and make recommendations for improving the current *Campylobacter* surveillance strategies in England.

## Methods

The enhanced surveillance study period ran from 7 June to 31 August and was divided into two phases. During phase 1 (7–30 June), all diagnostic laboratories in England were encouraged via the publication of a briefing note to send all *Campylobacter* isolates recovered from symptomatic patients to the GBRU for microbiological characterization using WGS. During phase 2 (5 July–31 August), a tiered approach was implemented, with laboratories requested to refer a specific number of randomly selected isolates each week. This number was based on their average annual detections of *Campylobacter* spp. (Table 1). This approach balanced the processing capacity of GBRU and associated WGS costs while maintaining the largest representative sample of isolates from across the country as could be accommodated within the period of enhanced surveillance.

**Table 1.** Maximum number of isolates according to tier to be referred to the GBRU each week during phase 2 (5 July–31 August) of the *Campylobacter* enhanced surveillance study

Laboratory tier category	Average laboratory confirmed *Campylobacter* spp. reported to SGSS each year (average 2017–**2019)**	Maximum no. of randomly selected isolates to be referred to GBRU per week
1	≤300	3
2	301–500	6
3	501–750	9
4	751–1,000	12
5	≥1,000	15

Isolates of *Campylobacter* spp. received by GBRU on Amies charcoal swabs were cultured on 5% Columbia blood agar, incubated at 37–42 °C for 24 h under microaerophilic conditions (5% O_2_, 5% CO_2_, 3% _H2_, 87% N_2_) using a Don Whitley M45 Microaerophilic Workstation. Only those isolates identified as viable, pure cultures following incubation were taken forwards for WGS. Genomic DNA was recovered from isolates using a QIAGEN QIAsymphony SP and DSP Viral/Pathogen Midi Kit, fragmented and tagged for multiplexing with Nextera XT DNA Sample Preparation Kits, followed by rapid run, paired-end sequencing on an Illumina HiSeq 2500 platform to produce 100 bp reads (Illumina, Cambridge, UK).

The FASTQ reads were quality trimmed using Trimmomatic (v0.27) with post-trim yields ranging from 56 to 840 megabase pairs (mbp) with a mean of 401 mbp. Species identification and purity were confirmed using kmer ID (https://github.com/ukhsa-collaboration/kmerid). The 7-loci multilocus sequencing type (MLST) was determined using MOST, a modified MLST typing tool based on short read sequencing [18]. Read mapping via the MOST tool to the 7 MLST genes provided further quality metrics, such as the percentage of non-consensus bases to the reference genes. A sample is considered to be mixed with more than one sequence type (ST) if this value is greater than 15%.

Antimicrobial-resistant (AMR) determinants inferring reduced susceptibility to erythromycin (macrolide), ciprofloxacin (fluoroquinolone), gentamicin and streptomycin (aminoglycosides), as well as tetracycline, were sought using ‘Genefinder’, a customized algorithm that uses Bowtie2 (v2.2.5) to map reads to a set of reference sequences and Samtools (v0.1.19) to generate an mpileup file (https://github.com/phe-bioinformatics/gene_finder). AMR determinants are considered to be detected if coverage of the reference gene by the sample’s reads is 100%, and the similarity to the reference gene is equal to or greater than 91%. Furthermore, there should be at least a 20 times average coverage of the reference gene.

SnapperDB [19] is software used to store single nucleotide polymorphism (SNP) variants, relative to a reference, in a database for each isolate that has undergone WGS and has at least an average coverage of 30 times the reference genome. Pairwise differences between isolates’ variants are calculated and stored in a distance matrix. Single-linkage hierarchical clustering is performed on this matrix at seven different SNP distance thresholds (250, 100, 50, 25, 10, 5 and 0), which results in a SNP address; this is a profile that describes the population structure. UKHSA routinely performs single-linkage hierarchical clustering on its SNP-typing data for other gastrointestinal pathogens (*Escherichia coli*, *Salmonella* and *Listeria monocytogenes*) to aid in outbreak detection and investigation.

The 5-SNP clustering level (referred to in this study as t5) was used in this study to group isolates together that have a common recent ancestor. This strategy was previously used in an enhanced molecular-based surveillance and source attribution study from which the authors inferred isolates in the same t5 group could be from a common source [20].

## Results

**Fig. 1.**
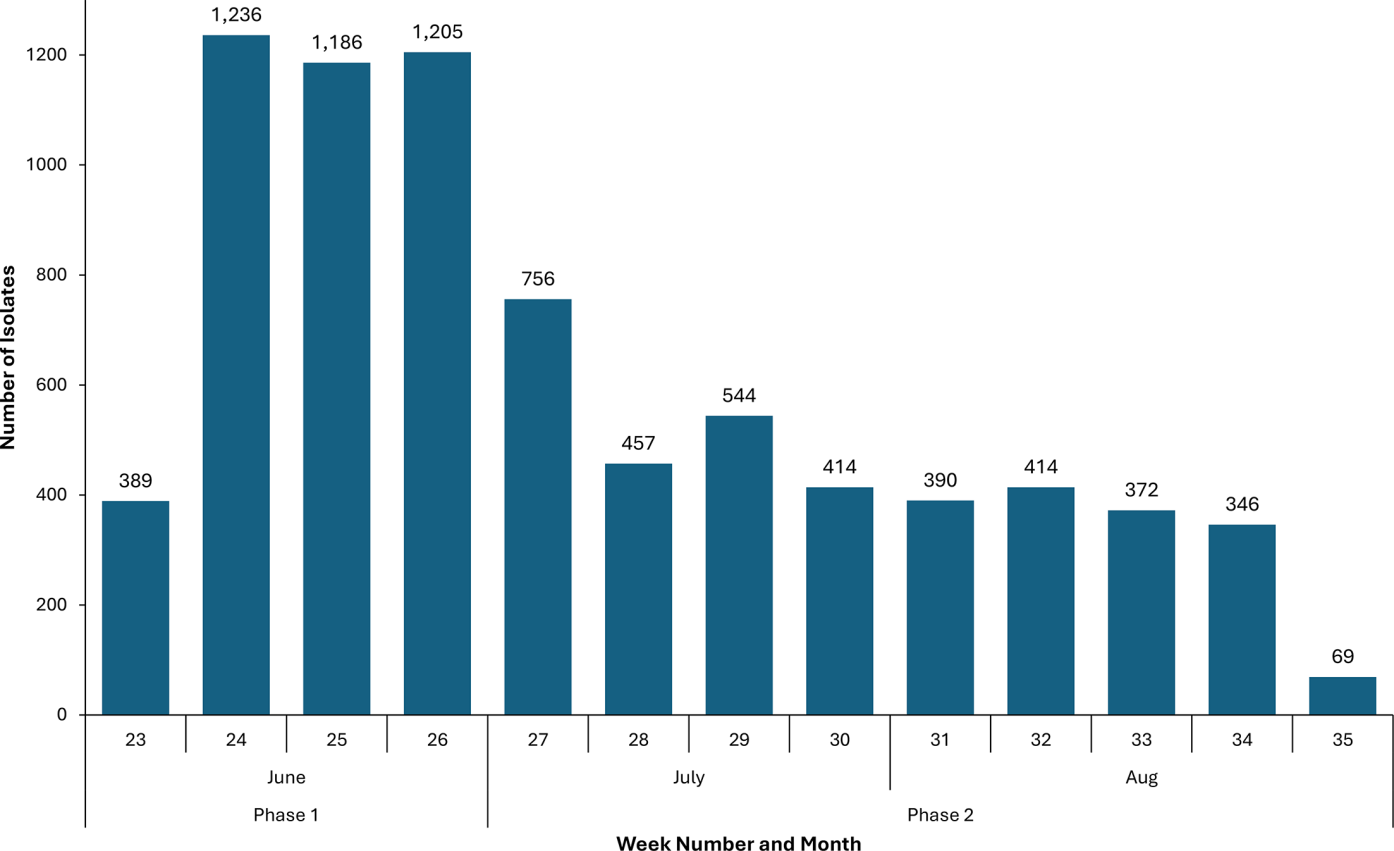
Isolates submitted per week to the GBRU during phase 1 and phase 2 (7 June–31 August 2021) of the *Campylobacter* Enhanced Surveillance Programme in England (*n*=7,778).

During the period of the UKHSA *Campylobacter* Enhanced Surveillance Programme, a total of 7,778 isolates from human cases with infection were received from microbiology laboratories across all nine UKHSA regions (East Midlands, East of England, London, North East, North West, South East, South West, West Midlands, Yorkshire and Humber) (Fig. 1). During phase 1 of the study (June 2021), GBRU received 51.6% of the total number of isolates (4,016/7,778). The highest weekly number of isolates (*n*=1236) was submitted during week 24 in June 2021. Subsequently, during phase 2, this number declined as laboratories were requested to submit only a proportion of *Campylobacter* spp. isolates. In July and August 2021, 27.9% (2,171/7,778) and 20.5% (1,591/7,778) of the total number of isolates were received by GBRU, respectively. Over the entire enhanced surveillance period, more than 1,000 samples were submitted by laboratories in the South West (*n*=1,321, 17.0%), South East (*n*=1,247, 16.0%), Yorkshire and Humber (*n*=1,163, 15.0%) and North West (1,115, *n*=14.3%) regions (Table 2).

**Table 2.** Number (percentage) of human *Campylobacter* species isolates submitted by laboratories in the nine UKHSA regions (*n*=7,778)

Region	Total no. of isolates (% of total)	No. of isolates identified					
		*C. jejuni*	*C. coli*	*C. lari*	*C. upsaliensis*	Mixture of *Campylobacter* spp*.* and/or strains	Contaminated/non-viable (% of regional total)
East Midlands	570 (7.3)	425	41	2	0	20	82 (14.4)
East of England	429 (5.5)	251	29	0	2	38	109 (25.4)
London	631 (8.1)	414	40	0	0	19	158 (25.0)
North East	481 (6.2)	330	32	1	1	31	86 (17.9)
North West	1,115 (14.3)	833	70	5	1	30	176 (15.8)
South East	1,247 (16.0)	795	67	2	0	27	356 (28.5)
South West	1,321 (17.0)	936	83	5	0	29	268 (20.3)
West Midlands	821 (10.6)	521	51	1	1	13	234 (28.5)
Yorkshire and Humber	1,163 (15.0)	787	78	5	4	28	261 (22.4)
Total	7,778	5,292	491	21	9	235	1,730 (22.2)

Of the 7,778 samples received, 6,048 (77.8%) were found to be viable and non-contaminated upon receipt and could, therefore, be taken forward for WGS, 1,230 (15.8%) were received as contaminated samples which could not be purified with the limited allocated laboratory resources available during the study period and the remaining 500 samples (6.4%) were non-viable upon receipt (Table 2).

### Species identification from WGS by kmer

Of the 6,048 cultures of *Campylobacter* spp. taken forward for species identification and typing by WGS, *C. jejuni* was the most commonly identified species, accounting for 87.5% (5,292/6,048) of isolates, followed by *C. coli*, accounting for 8.1% (491/6,048). Only 0.3% (21/6,048) were identified as *Campylobacter lari*, 0.1% (9/6,048) were identified as *Campylobacter upsaliensis* and the remaining 3.9% (235/6,048) were identified as mixed cultures of *Campylobacter* spp.

**Fig. 2.**
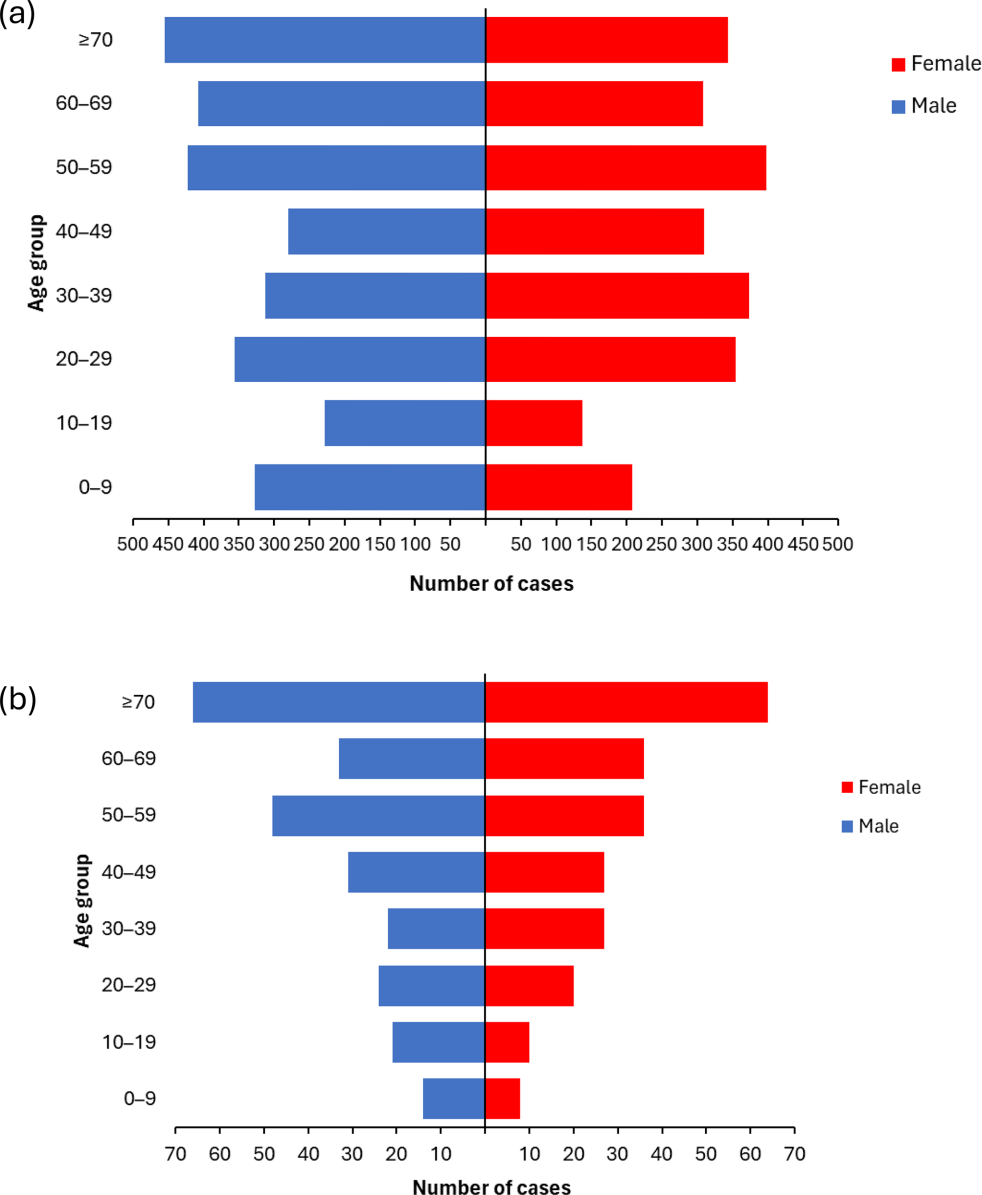
(a) Distribution of *C. jejuni* cases by age and sex (*n*=5,219) (b) Distribution of *C. coli* cases by age and sex (*n*=487).

Of the 5,292 *C. jejuni* isolates, age and sex data were available for 5,219 (98.6%), of which just over half were male (*n*=2,788, 53.4%). There was good representation of all age groups for *C. jejuni* isolates (Fig. 2a). Of the 491 *C. coli* isolates, age and sex data were available for 487 (99.2%) patients; again, more than half were from patients who were male (*n*=259, 52.7%). *C. coli* isolates tended to be from older patients (median age: 55 years) compared to *C. jejuni* isolates (median age: 44 years), and the age group with the highest number of isolates for *C. coli* was ≥70 years (*n*=130, 26.5%) (Fig. 2b).

### Multilocus sequencing type

**Fig. 3.**
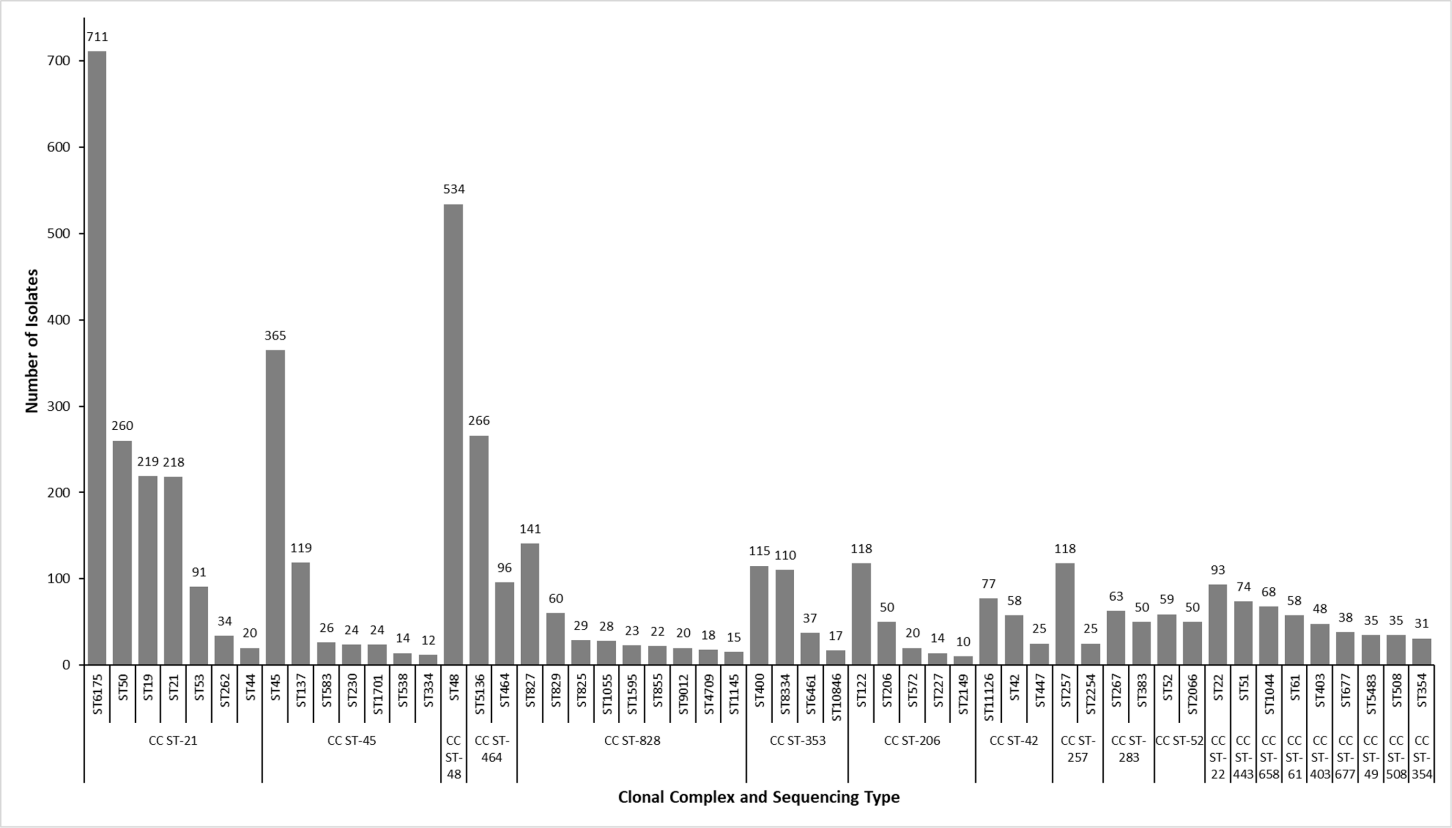
Twenty most common *Campylobacter* MLST clonal complexes and associated sequence types (minimum 10 isolates) identified during the study.

MLST was determined for 5,783 isolates of *Campylobacter* (5,292 isolates of *C. jejuni* and 491 isolates of *C. coli*), of which 5,308/5,783 (91.8%) could be grouped into 1 of 37 different MLST clonal complexes (CCs). The CC ST-21 was the largest of these, representing 1,586/5,308 (29.9%) isolates, followed by CC ST-45, representing 11.4% (*n*=604/5,308), and CC ST-48, representing 10.5% (*n*=558/5,308), while the remaining 34 CCs contained less than 500 isolates each. The 20 most commonly identified MLST CCs and associated STs with at least 10 isolates are shown in Fig. 3.

Overall, 165/5,783 (2.9%) isolates were assigned a novel uncharacterized ST profile at the time typing was undertaken, while the remaining 5618/5,783 (97.1%) were assigned one of 273 characterized STs, of which the three most common were ST6175 (711 isolates), ST48 (534 isolates) and ST45 (365 isolates). There were fewer than 300 isolates in each of the remaining 270 characterized STs (Fig. 3), and 112/273 (41.0%) characterized STs were represented by a single isolate and, therefore, appeared to be sporadic.

### SNP typing

SNP typing was successfully completed for 5,279/5,783 (91.3%) isolates of *Campylobacter* identified as either *C. jejuni* or *C. coli,* while the remaining 504/5,783 (8.7%) isolates could not be SNP-typed on a first attempt due to the poor quality of sequencing data generated.

**Fig. 4.**
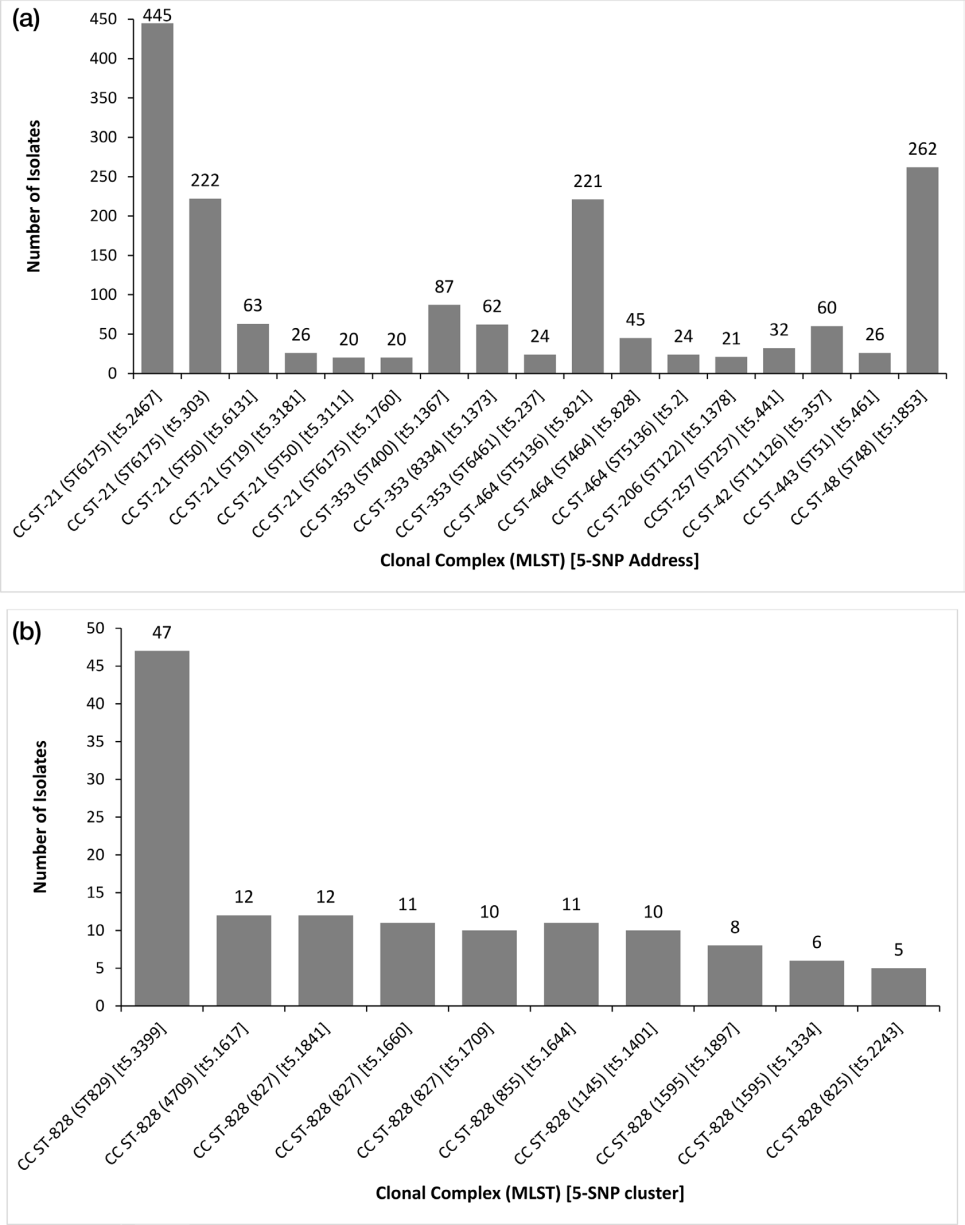
(a) *C. jejuni* 5-SNP clusters identified during the study with a minimum of 20 isolates, by MLST clonal complex and associated sequence types. (b) *C. coli* 5-SNP clusters identified during the study with a minimum of five isolates by MLST clonal complex and associated sequence types.

There were 4,838 isolates of *C. jejuni* successfully SNP-typed that fell into one of 1,846 5-SNP clusters. The four largest 5-SNP clusters (notation of ‘t5’ followed by a number is the 5-SNP cluster designated identifier), associated with the larger CCs and ST groups (ST6175 t5 : 2467, ST48 t5 : 1853, ST6175 t5 : 303 and ST5136 t5 : 821) accounted for 23.8% (*n*=1150/4,838) of *C. jejuni* isolates that could be SNP typed (Fig. 4a). Over half (56.5%) of all *C. jejuni* isolates successfully SNP-typed (*n*=2,734/4,838) could be assigned to one of 1,800 5-SNP clusters, each cluster containing 10 or fewer isolates, and 28.4% (*n*=1,375/4,838) appeared to be sporadic, each isolate assigned a unique SNP address at the 5-SNP level.

There were 441 isolates of *C. coli* successfully SNP-typed that fell into one of 237 5-SNP clusters. The largest 5-SNP cluster, (ST829 t5.3399) comprised 9.6% (*n*=47/441) of isolates of *C. coli* that could be SNP typed, while the remaining clusters each contained 12 isolates or less (Fig. 4b). Over a half (71.2%) of all *C. coli* successfully SNP typed (*n*=314/441) could be assigned to one of 228 5-SNP clusters, each cluster containing fewer than five isolates, and 39.5% (*n*=174/441) appeared to be sporadic, each isolate falling into a different single 5-SNP cluster.

### Detection of known antimicrobial resistance determinants in isolates of *C. jejuni* and *C. coli*

**Fig. 5.**
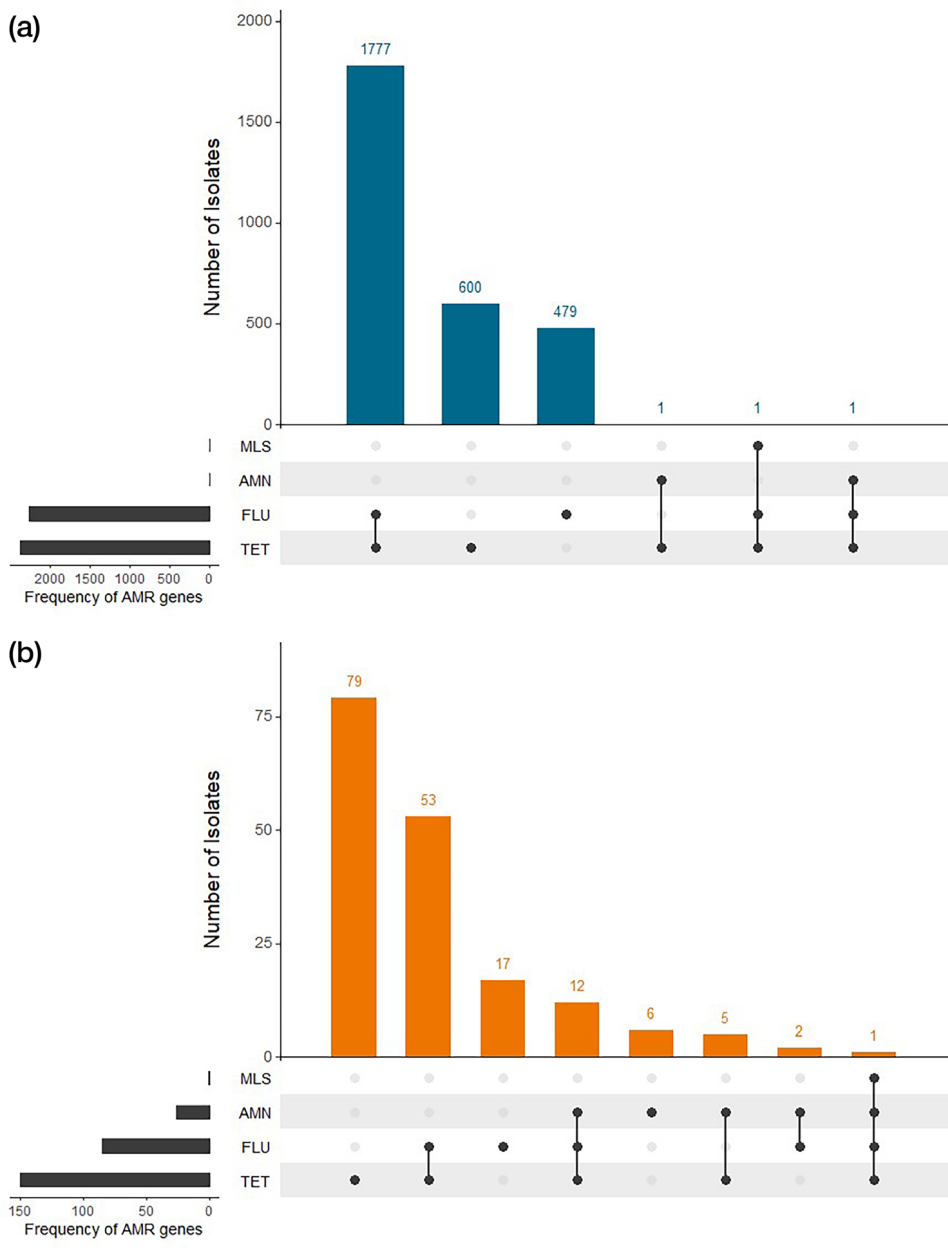
(a) Antimicrobial resistance profiles of *C. jejuni* isolates for the antibiotic classes macrolides (MLS), aminoglycosides (AMN), fluoroquinolones (FLU) and tetracyclines (TET). The number of isolates identified with single or combinations of resistance genes is shown in the bar chart at the top, and the dot matrix represents single (dots) or combinations (connected dots) of resistance genes. The frequency of the presence of each resistance gene is indicated in the bar chart on the left. (b) Antimicrobial resistance profiles of C*. coli* isolates for the antibiotic classes macrolides (MLS), aminoglycosides (AMN), fluoroquinolones (FLU) and tetracyclines (TET). The number of isolates identified with single or combinations of resistance genes is shown in the bar chart at the top, and the dot matrix represents single (dots) or combinations (connected dots) of resistance genes. The frequency of the presence of each resistance gene is indicated in the bar chart on the left.

Using analysis of WGS data to detect the presence of known AMR determinants to five antibiotics (ciprofloxacin, tetracycline, erythromycin, gentamicin and streptomycin) belonging to four different classes (fluoroquinolones, tetracyclines, macrolides and aminoglycosides), out of 5,292 isolates of *C. jejuni*, 42.7% (*n*=2,258/5,292) had the gyrA_CJ[86:T-I] mutation inferring resistance to ciprofloxacin (fluoroquinolone) and 45.0% (*n*=2,380/5,292) carried the tet(O) gene inferring resistance to tetracycline (tetracyclines). Less than 0.1% (*n*=2/5,292) contained the ant(6)-Ia (aadE) gene inferring resistance to streptomycin (aminoglycoside) or the 23 s [2075:A-G] mutation (*n*=1/5,292) inferring resistance to erythromycin (macrolide). The most common AMR profile was associated with both ciprofloxacin and tetracycline resistance (*n*=1,777/5,292, 33.8%), followed by isolates harbouring resistance to only tetracycline (*n*=600/5,292, 11.3%) and ciprofloxacin (*n*=479/5,292, 9.1%) (Fig. 5a). Less than 0.1% (2/5,292) of *C. jejuni* isolates were designated multi-drug resistant (MDR) carrying AMR determinants to at least three out of four different classes of antibiotics (macrolide, fluoroquinolone, aminoglycoside and tetracycline); the first being an isolate of ST2116 recovered from a patient in the 0–9 years of age category that harboured AMR determinants to fluoroquinolone (ciprofloxacin), macrolide (erythromycin) and tetracycline; and the second was an isolate of ST7991 recovered from a patient in the 30–39 years of age category that harboured AMR determinants to aminoglycosides (streptomycin), fluoroquinolone (ciprofloxacin) and tetracycline. Of the seven most common *C. jejuni* STs, each with over 200 isolates identified in this study, over 96.0% of ST6175 and ST5136 isolates and 27.0% of ST50 isolates were predicted to be susceptible to both ciprofloxacin (fluoroquinolone) and tetracycline. Just over 34.0% of isolates of ST19 and ST21 were predicted to be susceptible to ciprofloxacin (fluoroquinolone) alone, while just over 67.0% of ST48 isolates were predicted to be susceptible to only tetracycline. Those isolates of ST45 identified in this study were rarely predicted to be susceptible to these antimicrobial agents (Table 3).

**Table 3.** Number of *C. jejuni* isolates with assigned MLST clonal complexes and associated sequence types and percentage of identified known antimicrobial resistance determinants

MLST						
Clonal complex	Sequence type	Fluoroquinolones	Tetracyclines	Aminoglycoside	Macrolide	Fluoroquinolone and tetracycline
ST-21	ST6175 (*n*=711)	0.0%	2.5%	0.0%	0.0%	96.8%
	ST50 (*n*=260)	11.2%	8.5%	0.0%	0.0%	27.3%
	ST19 (*n*=219)	34.7%	0.5%	0.0%	0.0%	1.4%
	ST21 (*n*=218)	34.9%	3.7%	0.0%	0.0%	4.1%
ST-45	ST45 (*n*=365)	2.5%	0.6%	0.0%	0.0%	0.0%
ST-48	ST48 (*n*=534)	1.5%	67.8%	0.0%	0.0%	0.9%
ST-464	ST5136 (*n*=266)	0.0%	1.9%	0.0%	0.0%	97.0%

From analysis of WGS data generated from the 491 isolates of *C. coli*, 30.5% (*n*=150/491) carried mutations in the tet(O) gene, 17.3% (*n*=85/491) carried the gyrA_CJ[86:T-I] mutation and 5.3% (*n*=26/491) contained the aadK gene. Only 0.2% (*n*=1/491) carried the 23 s [2075:A-G] mutation. The majority of isolates carried mutations for only tetracycline resistance (*n*=79, 16.1%) or ciprofloxacin and tetracycline resistance (*n*=53, 10.8%) (Fig. 5b). Thirteen *C. coli* isolates were designated MDR, of which 12 (2.4%) harboured resistance determinants for aminoglycosides (streptomycin), fluoroquinolone (ciprofloxacin) and tetracycline, and 1 (<0.1%) had inferred resistance to all four antibiotic classes.

## Discussion

Historically, public health surveillance of campylobacteriosis in England has been hampered by the overwhelming case numbers and the limitations of the phenotypic typing methods (e.g. serotyping and phage typing) prior to the introduction of WGS [21,22]. The lack of a robust typing approach led to the perception that outbreaks of campylobacteriosis were rare and that investment in a more comprehensive framework for surveillance would have limited value [23]. The advent of WGS has provided public health agencies with a robust typing scheme for investigating outbreaks caused by *C. jejuni* and *C. coli* and monitoring trends. However, the high number of cases remains challenging from both an economic and operational perspective.

The widespread move towards implementation and reliance on local enteric PCR panels in diagnostic laboratories for the detection of *Campylobacter* spp. rather than a classical culture approach, coupled with limited resources and associated costs to implement recovery of *Campylobacter* spp*.* from stool specimens at these laboratories, is likely a contributing factor in varying success to our requests to submit isolates during the study period from different regional areas.

In this enhanced laboratory surveillance of *Campylobacter* spp. in England, 7,778 isolates from human cases with infection were received into GBRU for identification and typing. This represents a significant increase in referrals from less than 2.0% of annual detections reported by diagnostic laboratories to SGSS in previous years, to ~45.0% during this study period, thereby increasing our understanding of the molecular epidemiology during this period by describing the circulating strains and AMR profiles in England at the peak of activity during the summer months of 2021. Furthermore, this provides a baseline dataset against which future data can be compared year on year, providing additional insights into epidemiological trends, particularly the most common strains during the peak of the season. Phase 1 of this enhanced laboratory surveillance of *Campylobacter* highlighted several operational challenges in attempting to culture and type significantly large numbers of isolates in real time within our laboratory. Some of the challenges were resolved by moving to the tiered approach in phase 2, although the smaller sample size may have impacted the genetic diversity of isolates received during that phase of the study. A key challenge was the limited capacity within microaerophilic incubators, such that there was no additional capacity to attempt to purify any of those isolates received as contaminated or mixed cultures. It was noted that the region with the highest proportion of viable and non-contaminated cultures was the East Midlands (85.6%), while conversely, those regions with the lowest proportion were the South West and West Midlands (71.5%). Regional discrepancies were also noticed for those samples identified as mixed cultures, with laboratories in the North East and the East of England, which were, respectively, twice and three times more likely to submit mixed samples. For future surveillance efforts, this challenge could be mitigated through engagement with referring laboratories to ensure awareness of best practices, and any increase in contaminated or non-viable isolates is detected and addressed quickly.

There were also limitations with regard to the number of samples from which genomic DNA could feasibly be extracted in a single working day using QIAGEN QIAsymphony platforms and sequenced on an Illumina HiSeq 2500. These limitations, which should be considered in any future enhanced surveillance work, created bottlenecks in processing at the GBRU and significant delays in reporting the typing results within our published laboratory turnaround times. Therefore, while identified clusters were considered for epidemiological investigation, the delays meant that real-time collection of epidemiological information through case questionnaires was not possible during this study.

Despite these operational challenges, there was a good response from the regional UKHSA laboratories and the NHS network to the communication requesting submission of isolates to GBRU for sequencing from all regions, providing comprehensive coverage across England.

In line with previous studies reporting similar trends in cases of human infections [24,25], most isolates typed during this enhanced surveillance were identified as *C. jejuni,* followed secondly by *C. coli*, and these proportions were consistent across the regions. Age-sex distributions were consistent across each region, and the proportion of male to female cases was consistent between the two major species. The age distribution of referred *C. jejuni* isolates was similar to the age distribution of positive *Campylobacter* laboratory reports notified to UKHSA in 2022 [26]. However, there was a higher proportion of referred *C. coli* isolates from older age groups. Further investigation into whether this reflects a true difference in the epidemiology of *C. coli* or whether it was due to the sampling approach taken is needed.

Using WGS to derive MLST was proven to be a robust approach to typing *C. jejuni* and *C. coli*, as all isolates were typed, with 2.9% being novel, previously unknown types. Global studies consistently report CC ST-21 among the most common types of * C. jejuni* [27]. As was the case in this data set, previous typing data obtained on isolates of *C. jejuni* recovered from cases of human infections in England, in North Tyneside and Oxfordshire, between October 2015 and September 2018 [20], showed that CC ST-21 was the most commonly isolated ST. However, while ST6175 was identified as the most common ST within CC ST-21 in this 2021 enhanced surveillance study, it was detected in <1.0% of all *C. jejuni* recovered from symptomatic cases of human infection in the North Tyneside and Oxfordshire study [20]. Conversely, although ST50 was the most common ST identified in the North Tyneside and Oxfordshire study, it was the fifth most common ST in this 2021 dataset. This suggests a changing population dynamic of * C. jejuni* causing infections in England, with the rise and fall of different types perhaps linked to fluctuations in the dominant types in the animal reservoir, most likely changes in poultry flock colonization, since contaminated chicken is widely accepted as the main vehicle for infection in the UK. It may also reflect changes in the exposure risks and/or transmission routes, such as travel or imported produce. This highlights the need for consistent surveillance over extended periods and typing of isolates from animals, food and human sources in a one-health approach.

SNP typing was shown to be a rapid method applicable to clustering the majority of isolates of *C. jejuni* and *C. coli* from cases of human infections in this study. Almost a quarter of all *C. jejuni* were grouped into only four 5-SNP clusters within the clonal complexes ST-21, ST-48 and ST-464. As consumption of raw or undercooked poultry meat contaminated with *Campylobacter* spp. has been recognized as one of the primary routes of human disease, these clusters potentially represent large retail poultry reservoirs or isolates derived from common ancestral flocks. Further work should be undertaken to compare the clustering of isolates in this dataset using a SNP typing approach versus core genome MLST, to evaluate the impact of recombination events in *Campylobacter* genomes on clustering.

Using established bioinformatic methods [28], reduced susceptibility to ciprofloxacin (fluoroquinolone) and tetracycline was predicted from isolates of *C. jejuni* successfully assayed in this study (42.7% and 45.0%, respectively). These levels are similar to those seen in recent studies conducted on clinical isolates in 2015–2018, indicating reduced susceptibility to ciprofloxacin (fluoroquinolone) in 45.1% and to tetracycline in 42.8% of *C. jejuni* from symptomatic cases [20]. Similarly, reduced susceptibility to erythromycin (macrolide) was only detected in <0.1% (*n*=1/5,292) of *C. jejuni* isolates in this enhanced laboratory surveillance study and in only 0.4% of clinical isolates in 2015–2018 [20]. This suggests that the prevalence of susceptibility to these three antimicrobial agents (ciprofloxacin, tetracycline and erythromycin) in clinical isolates of *C. jejuni* remained relatively consistent over the 3-year period between 2018 and 2021.

It was noted that the proportion of ciprofloxacin-resistant isolates of *C. coli* was lower (17.3%, *n*=85/491) in this enhanced laboratory surveillance study compared to those levels reported in previous studies, which ran from 2015 to 2018 (36.6%) [20]. This may be an artefact of the sampling scope and timeframe in this study (i.e. during the summer months June–August) in comparison to an annual sentinel surveillance strategy across a 3-year period or the prevalence of particular ciprofloxacin-susceptible lineages of * C. coli* sampled during the restricted timeframe of the 2021 dataset and requires further investigation. This potentially highlights the need for year-round surveillance of AMR in *Campylobacter* rather than reliance on a snapshot across only a 3-month timeframe and that monitoring of AMR in *Campylobacter* should be made separately for *C. jejuni* and *C. coli*.

## Conclusion

The results of this enhanced laboratory surveillance study for *Campylobacter* in England provide valuable information in understanding the latest molecular epidemiology and AMR profiles of *Campylobacter* causing gastrointestinal disease. It demonstrates that WGS-enabled identification of genetically related clusters of *Campylobacter* cases in almost real time is possible and shows potential for monitoring of AMR. However, unlocking its full potential requires referral of sufficient and representative isolates to GBRU with parallel epidemiological data collection. Furthermore, this study highlights the benefit of maintaining this surveillance throughout the year to account for potential seasonal variation. Further work is ongoing to statistically determine the minimum number of isolates and sampling frame required to enable detection of major clusters and monitor changes in predominant STs and AMR in England, as well as considering the cost-effectiveness of the different strategies. However, at this time, we recommend a minimum of 20 isolates of *Campylobacter* spp. per month should be referred from cases of human infections by each of the regional UKHSA laboratories to the GBRU for typing by WGS. This recommendation attempts to balance both the financial and logistical impacts of real-time WGS typing at a national level for a pathogen as prevalent as *Campylobacter* until further analyses have been completed to determine regional representative proportions of isolates that would be required to achieve a cost-effective and meaningful ongoing molecular surveillance strategy in England.

## Abbreviations

AMR: antimicrobial resistance
CCs: clonal complexes
COVID-19: coronavirus disease 2019
EU: European Union
GBRU: Gastrointestinal Bacteria Reference Unit
GBS: Guillain–Barré syndrome
mbp: megabase pairs
MDR: multi-drug resistant
MLST: multilocus sequencing type
SGSS: Second Generation Surveillance System
SNP: single nucleotide polymorphism
ST: sequence type
WGS: whole-genome sequencing
